# Skin and Bones: The Contribution of Skin Tone and Facial Structure to Racial Prototypicality Ratings

**DOI:** 10.1371/journal.pone.0041193

**Published:** 2012-07-18

**Authors:** Michael A. Strom, Leslie A. Zebrowitz, Shunan Zhang, P. Matthew Bronstad, Hoon Koo Lee

**Affiliations:** 1 Department of Psychology, Brandeis University, Waltham, Massachusetts, United States of America; 2 Department of Psychology, Yonsei University, Seoul, Korea; Tel Aviv University, Israel

## Abstract

Previous research reveals that a more ‘African’ appearance has significant social consequences, yielding more negative first impressions and harsher criminal sentencing of Black or White individuals. This study is the first to systematically assess the relative contribution of skin tone and facial metrics to White, Black, and Korean perceivers’ ratings of the racial prototypicality of faces from the same three groups. Our results revealed that the relative contribution of metrics and skin tone depended on both perceiver race and face race. White perceivers’ racial prototypicality ratings were less responsive to variations in skin tone than were Black or Korean perceivers’ ratings. White perceivers ratings’ also were more responsive to facial metrics than to skin tone, while the reverse was true for Black perceivers. Additionally, across all perceiver groups, skin tone had a more consistent impact than metrics on racial prototypicality ratings of White faces, with the reverse for Korean faces. For Black faces, the relative impact varied with perceiver race: skin tone had a more consistent impact than metrics for Black and Korean perceivers, with the reverse for White perceivers. These results have significant implications for predicting who will experience racial prototypicality biases and from whom.

## Introduction

Recent research provides strong evidence that responses to faces are influenced not only by their perceived racial category, but also by their phenotypic qualities regardless of category. Black or White individuals who are judged to have a more prototypically Black appearance elicit more stereotyped trait impressions [1], more negative associations [2], and harsher penalties in the criminal justice system [3], [4]. Indeed racial prototypicality sometimes has a stronger effect on social outcomes than racial category, perhaps because people try to avoid race bias but are unaware of the more subtle prototypicality bias [5]. Whereas significant effects of racial prototypicality have been well-documented, the question remains as to what facial qualities influence perceived racial prototypicality. The goal of the present research was to investigate the relative influence of skin tone and facial metrics on racial prototypicality ratings of White, Black, and Korean perceivers, and whether these effects vary with face race. Although we recognize that anthropologists and biologists question the validity of race as a scientific concept (e.g., Lewontin 1972), it is nevertheless, a widely accepted concept in folk psychology (Zuckerman 1990). While acknowledging that clear decisions on category membership are problematic, we use the ‘fuzzy’ category system of racial groups and the terms *White, Black,* and *Korean* to denote the physical appearance of target faces and the group identification of perceivers in this research.

Much of the research investigating the facial qualities involved in race perception has emphasized variations in skin tone. When simply asking participants to rate the importance of various facial features and skin color in determining the race of a target, skin color emerged as the most important cue [6]. Consistent with people’s ratings of the importance of skin tone, both Black and White perceivers categorize Black individuals according to their skin tone, as evidenced in an implicit recall measure of who said what when targets differed in skin tone, which paralleled the effects found when targets differed in race [7]. Using a similar paradigm, African-American children showed better memory of what story characters did when the stories paired light-skinned Black targets with positive traits and high status occupations and dark-skinned Black targets with negative traits and low status occupations [8]. Also, both Black and White perceivers reported more negative cultural beliefs about the traits of darker-skinned than lighter-skinned Black targets, with the reverse trend for positive beliefs [7]. Another study found that European Americans high in racial prejudice were faster to recognize the onset of anger and slower to recognize the offset of anger in schematic Black than White faces, when face race was manipulated solely by skin tone [9]. Although the foregoing studies did not assess perceived racial prototypicality per se, they do provide reason to believe that skin tone may be an important determinant of such judgments. Also, while the foregoing studies assessed Black and/or White perceivers responses to skin tone, there is evidence for a preference for light skin in Asian perceivers as well [10], suggesting that skin tone may be an important determinant of perceived racial prototypicality for Koreans as well as for Black and White Americans.

Although the results of the foregoing studies may reflect responses to skin tone, it is also possible that many reflect responses to facial structure. For example, the images conjured up when participants were instructed to think of darker- vs. lighter-skinned Black targets [7] may have differed on dimensions other than skin tone. Other research that has separated effects of skin tone and facial structure suggests that facial structure may be more important. A neuroimaging study found that regardless of whether Black faces had light or dark skin, they elicited higher amygdala activation (an indicator of emotional salience) in White viewers than did White faces, suggesting the importance of facial structure [11]. Similarly, other researchers found that observers’ evaluations of a perpetrator in a simulated news report did not differ whether given light, medium, or dark skin when facial structure was held constant [12]. In a more recent investigation, ratings of skin tone and racial prototypicality of grey scale facial images were collected using the lightness contrast illusion [13]. As predicted, when a grey scale image of a face that had been morphed to a mixed Black and White racial appearance was surrounded with Black faces, color ratings of the face were lighter than when it was surrounded with White faces. However, the face’s perceived racial prototypicality was not affected, suggesting that facial metrics may be the more influential cue to racial prototypicality. In contrast to the foregoing failures to find independent effects of skin tone on judgments of Black faces or Black-White morphs, another study found independent effects of both skin tone and face shape on racial categorization of Japanese and White faces [14].

The preceding research has several limitations. First, previous research has not systematically investigated effects of perceiver race on the relative influence of skin tone and facial structure. In many studies perceiver race is not reported, and in those that do report race, the large majority of participants were White, thereby precluding analyses to examine effects of perceiver race. Yet, there is reason to predict such effects. Anecdotal evidence regarding differential treatment of darker and lighter skinned Blacks within the African-American community [10] coupled with an injunction to be ‘color blind’ that is experienced by White Americans, but possibly not Koreans, suggests that racial prototypicality ratings of White perceivers may be less responsive to skin tone than those of Black or Korean perceivers. Indeed, this prediction is consistent with the research summarized above that used perceivers whose race was either unspecified or predominantly White [11]–[13] and found that facial structure trumped skin tone. In contrast, the one study of Japanese perceivers found an influence of both [14]. An additional limitation of the existing research is that it has not systematically investigated moderating effects of face race on the relative influence of skin tone and facial structure. However, it is noteworthy that the studies that found that facial structure was more important than skin tone examined faces that were completely or partially Black, whereas one that found that the two were equally important examined faces that were White or Japanese. A final limitation of the existing research vis a vis the aims of the present study is that many of the studies bear on processes other than racial prototypicality ratings.

The present study filled the aforementioned gaps in our understanding of racial prototypicality by achieving two aims. The first was to compare the relative contribution of skin tone and facial metrics to the racial prototypicality ratings of White, Black, and Korean perceivers. Based on the existing research we predicted that White perceivers would be more influenced by facial metrics than skin tone [11]–[13], whereas the two facial qualities would have relatively equal influence for Korean perceivers [14] and that Black perceivers may be more influenced by skin tone [10]. We further expected that White perceivers would be less influenced by skin tone than perceivers of other races. Our second aim was to determine whether the relative contributions of skin tone and facial metrics to racial prototypicality judgments differ across face race. It is difficult to make an informed prediction about this effect, since the literature has largely confounded perceiver and face race, focusing on White perceivers’ responses to Black faces, with only one study examining Asian perceivers’ responses to Asian and White faces. Achieving these two aims not only will inform our understanding of face perception, but it also has practical importance. As noted above, more African-looking White or Black defendants receive longer prison sentences [3], and more African-looking Black defendants convicted of murdering a White victim are more likely to receive the death penalty [4]. Our results will have significant implications for predicting who will experience racial prototypicality biases and from whom.

## Methods

### Ethics Statement

The research was conducted in accordance with the ethical principles for research involving human subjects expressed in the Declaration of Helsinki. The research protocol was approved by the Institutional Review Board at Brandeis University. Written informed consent was obtained from all participants.

### Participants

Thirty nine White American college undergraduates (17 males), 26 Black American college undergraduates (11 males), and 48 Korean college undergraduates (24 males) at a university in Seoul, Korea rated race-related appearance qualities and emotion expression of the target faces. White and Korean participants were randomly assigned to rate either male or female faces, while Black participants rated faces of both sexes with the order of face sex counterbalanced across participants. Thus, each face was rated by approximately 20 White participants, 26 Black participants, and 24 Korean participants. White participants received either $10 or course credit for their participation in a single session, and Korean raters received the equivalent of $10 in their local currency for participation in a single session. Black participants received $25 for their participation in two experimental sessions.

### Faces

There were 60 White facial images, 60 Black facial images, and 60 Korean facial images, with male and female faces equally represented, all of which had been used in a previous study [15]. Four criteria were used for target face selection: neutral expression, no head tilt, no glasses, and no facial hair. Faces were presented in color against a beige background. White facial images were selected from a variety of databases: University of Stirling PICS database, the AR face database [16], and yearbooks from an American high school and a university. The facial images of Black males were from a set of faces that had been used in a study that found significant effects of racial prototypicality on stereotyping [1]. These images had been selected by the authors from American high school yearbooks. The images of the Black female target faces were selected from the website http://americansingles.com by searching for Black females ages 18–25. Korean facial images were selected randomly from a Korean university yearbook with the constraint that they meet the four selection criteria listed above.

### Facial Metric Measurements

**Figure 1:**
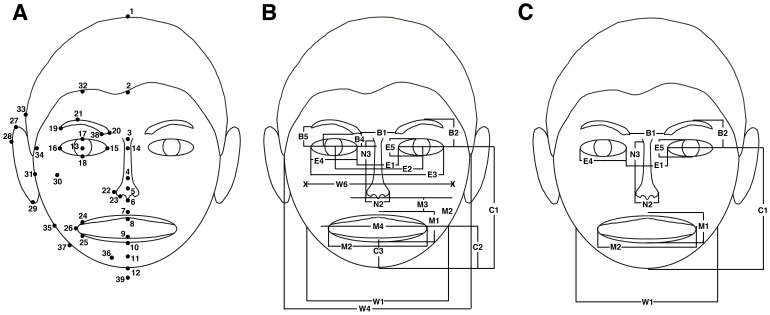
Facial metric measurements. Panel A shows location of points utilized for establishing facial metrics. When identical points were marked on the right and left side, only those on the person’s right side are indicated. Panel B shows location of the reliably measured facial metrics that were used in the discriminant function analysis. Panel C shows location of facial metrics that significantly discriminated different race faces and were used in the regression analyses.

Following the procedure in previous studies [17], [18], [19], in house software was used to mark 64 points on digitized images of each face viewed on a 21 inch PC monitor, from which facial metrics, normalized by interpupil distance, were computed using automatic procedures written in Visual Basic and Excel. This is a standard normalization technique to control for variations in distance from the camera [20]–[22]. Points marked by two research assistants (Figure 1, Panel A) yielded 21 facial metrics with acceptable inter-judge reliability (rs >7 for faces of all races and both genders), and these were used in subsequent analyses (Figure 1, Panel B). Evidence for the predictive validity of facial metrics derived through this procedure has been provided in previous research that input the metrics into connectionist models. For example, adult faces with metrics that resemble those of babies were rated as more babyfaced and as possessing more childlike traits [17]; normal faces with metrics that resemble those of anomalous faces were rated as less attractive and less healthy [17]; neutral expression White, Black, and Korean faces with metrics that resemble those of angry expression faces were rated as more hostile and less trustworthy [19].

### Skin Tone Ratings

All faces were rated on a 7-point scale assessing *skin tone* (very light skin color – very dark skin color). We used a subjective rating of skin tone rather than objective measures because, for our purposes, the contribution to prototypicality judgments of *perceived* differences in skin tone among faces that are all the same race is the most relevant variable. The standardized alpha for skin tone ratings was.92, averaged across ratings of male and female faces of each race by perceivers of each race.

### Racial Prototypicality Ratings

All faces were rated on 7-point appearance scales assessing: *Caucasian appearance* (not at all Caucasian/White – very Caucasian/White), *African appearance* (not at all African – very African), and *Asian appearance* (not at all Asian - very Asian). Standardized alphas averaged across ratings of male and female faces of each race by perceivers of each race were.83 for Caucasian appearance ratings,.85 for African appearance ratings, and 86 for Asian appearance ratings.

### Other Appearance Ratings

Three control appearance variables (attractiveness, babyfaceness, and smiling) were taken from ratings of the faces provided by the same participants for a previous study in which these ratings had shown acceptable reliability [15]. Although all faces were selected to have a neutral expression, a smile variable was created by dividing the number of times participants had identified the face as happy by the total number of participants (other expressions were not mentioned with sufficient frequency to create additional control variables). Attractiveness (unattractive – attractive) and babyfaceness (babyfaced – maturefaced) were rated on 7-point scales. Faces also were rated as to how masculine/feminine they looked, but these ratings were not used in the analyses.

### Korean Translation

All rating scales were translated into Korean by a native Korean speaker. A second native Korean speaker translated the Korean back into English, and these results were compared to the original English-language scales. For any discrepancies, the native Korean speakers were consulted to retranslate the scales so that the meaning in Korean was as close as possible to the meaning in English.

### Procedure

Perceivers viewed images and input responses on Pentium 4 personal computers with Windows XP and 19” CRT displays with 1280×1024 screen resolution. Raters sat within 36” of the monitors. MediaLab 2004.2.1 [23] was used to display images and collect ratings. Identical computers and monitors were purchased for data collection in Korea, and identical Medialab programs for presentation of stimuli with English or Korean instructions and rating scales were prepared in the Brandeis face perception lab. Faces were displayed until a rating was made, with a maximum duration of 5 seconds, after which the face disappeared and the rating scale remained until a rating was made. Faces of each race were rated first on prototypicality pertinent to that race (e.g. Black faces were rated first on African prototypicality), with the order of the other two prototypicality judgments counterbalanced. Perceivers were instructed to focus on rating each face according to how racially prototypical the features were. Although participants were instructed to focus on how racially prototypical the features were, the finding that perceived skin tone predicted those ratings over and above facial metrics indicates that they captured racial prototypicality more broadly. The order in which Black, White, and Korean faces were rated was counterbalanced across raters.

## Results

### Discriminating Facial Metrics and Perceived Skin Tone

To select a set of facial metrics most likely to influence prototypicality ratings, we entered the 21 reliable facial metrics simultaneously into discriminant function analyses comparing two races at a time to identify metrics that objectively discriminated between faces of different races. The discriminant analyses were statistically significant for each of the racial comparisons: White and Black faces’ Wilks’ Lambda = 24, Chi-square = 162.92, p<001; White and Korean faces’ Wilks’ Lambda = 18, Chi-square = 197.64, p<001; and Black and Korean faces’ Wilks’ Lambda = 10, Chi-square = 262.36, p<001. A total of eleven facial metrics objectively discriminated at least two groups of faces (Figure 1, Panel C). The standardized coefficients for each metric are shown in Table 1. The means and standard deviations of the facial metrics that objectively discriminated the races as well as the skin tone ratings are shown in Table 2. Compared to Black faces, White faces had wider jaws, narrower noses, thinner lips, lower eyebrows, and longer chin to pupil height. Compared to Korean faces, White faces had larger vertical eye height (distance from the upper to lower eyelid), smaller jaw width, smaller eye separation, smaller eyebrow separation, wider mouths, thinner lips, and lower eyebrows. Compared to Korean faces, Black faces had narrower jaws, smaller eye separation, wider and shorter noses, smaller eyebrow separation, larger horizontal eye width, and larger mouth width.

**Table 1:** Objectively Discriminating Facial Metrics.

Metric Label	Metric Name	Standardized Coefficients with t-values (in parentheses)		
		White vs. Black Faces	White vs. Korean Faces	Black vs. Korean Faces
E5	Vertical Eye height		.54 (7.07**)	
W1	Jaw width	.53 (4.36**)	−.53 (7.76**)	−.97 (11.03**)
E1	Eye separation		−.49 (12.12**)	−.46 (13.13**)
N2	Nose width	−.85 (9.68**)		.67 (7.00**)
N3	Nose length			−.62 (7.02*)
B1	Eyebrow separation		−.38 (6.67**)	−.29 (4.46**)
E4	Horizontal eye width			.36 (9.06**)
M0	Mouth width		.34 (2.09*)	.34 (5.97**)
M1	Lip thickness	−.57 (9.94**)	−.31 (5.54**)	
B2	Eyebrow height	−.34 (3.03*)	−.23 (4.50**)	
C1	Chin to pupil height	.49 (3.79**)		

*Note.* Positive values for standardized coefficients indicate higher values for White faces, and for Black faces in the Black vs. Korean analysis.

* *p*<.05;

** *p*<001.

**Table 2:** Descriptives for Facial Quality Predictors.^a^

Facial Quality			Face Race					
Name	MetricLabel		White		Black		Korean	
			M	SD	M	SD	M	SD
Vertical Eye height	E5		.17	.02	.*16*	.02	.14	.02
Jaw width	W1		1.87	.11	1.77	.14	2.03	.12
Eye separation	E1		.51	.03	.51	.03	.59	.03
Nose width	N2		.57	.05	.66	.06	.60	.04
Nose length	N3		.*72*	.07	.68	.06	.75	.05
Eyebrow separation	B1		.37	.10	.41	.11	.50	.11
Horizontal eye width	E4		.*81*	.05	.84	.05	.76	.05
Mouth width	M0		.84	.11	.88	.08	.80	.07
Lip thickness	M1		.25	.06	.34	.05	.30	.04
Eyebrow height	B2		.36	.05	.39	.05	.40	.04
Chin to pupil height	C1		1.84	.12	1.76	.13	1.87	.11
**Skin Tone**								
White Perceivers		3.14^a^		1.00	4.62^b^	1.35	3.43^a^	.90
Black Perceivers		2.29^a^		.83	4.21^b^	1.54	3.64^c^	.96
Korean Perceivers		3.24^a^		1.20	4.42^b^	.99	3.86^c^	1.06

a *Note*. Means for facial metrics that did not discriminate a particular race from the others are shown in italics. Skin tone means with different superscripts within each perceiver group differ at *p<*01 or better.

A 3 (face race) × 3 (rater race) analysis of variance on skin tone ratings revealed a significant effect of face race, *F* (2, 177) = 39.98, *p*<001, reflecting a tendency for Black faces to be rated as darker skinned than both White and Korean faces, *ps* <001, and for Korean faces to be rated as darker than White faces, *p*<001. A significant effect of rater race, *F* (2, 177) = 18.83, *p*<001, reflected a tendency for both White and Korean perceivers to give darker skin tone ratings than did Black perceivers, *ps* = 001. There also was a significant rater race by face race interaction, *F* (4, 177) = 10.44, *p*<001. Planned comparisons revealed that the overall tendency to rate Black faces as darker than Korean faces which in turn were rated as darker than White faces was significant for perceivers of all races with the exception of White perceivers ratings of White and Korean faces, *p* = .15 (see Table 2).

### Effects of Skin Tone vs. Facial Metrics on Perceived Racial Prototypicality

Table 3 shows the mean racial prototypicality ratings of White, Black, and Korean faces by raters of each race. Not surprisingly, perceivers of all races rated White faces as higher in Caucasian- than African- or Asian-prototypicality, Black faces as higher in African- than Caucasian-or Asian-prototypicality, and Korean faces as higher in Asian- than Caucasian- or African- prototoypicality. Also, Caucasian prototypicality ratings were higher for White than Black or Korean faces, African ratings were higher for Black than White or Korean faces, and Asian ratings were higher for Korean than White or Black faces.

**Table 3:** Descriptives for Racial Prototypicality Ratings.^a^

		Face Race					
PerceiverRace	RacialPrototypicality	White		Black		Korean	
		M	SD	M	SD	M	SD
White	Caucasian	5.02^a^	.80	2.68^c^	.74	3.12^d^	.54
	African	2.19^b^	.43	4.99^a^	.75	1.76^c^	.30
	Asian	2.26^b^	.38	2.78^c^	.83	5.56^a^	.52
Black	Caucasian	5.25^a^	.91	2.41^b^	.72	2.52^b^	.74
	African	2.23^b^	.66	4.96^a^	.99	2.37^b^	.50
	Asian	2.20^b^	.63	2.04^c^	.51	5.44^a^	.59
Korean	Caucasian	4.56^a^	.96	3.00^b^	1.01	3.10^b^	.80
	African	2.98^b^	.80	5.00^a^	1.03	3.07^b^	.78
	Asian	3.33^c^	.78	3.01^b^	.86	4.82^a^	.76

a *Note*. Within each perceiver race, face race effects (row means) with different superscripts and racial prototypicality effects (column means) with different subscripts differ at *p<*05 or better.

To examine facial qualities than influenced the perceived racial prototypicality of faces *within* each race, we performed a series of regression analyses predicting racial prototypicality ratings using face as the unit of analysis, which was justified by high inter-rater reliabilities for the face ratings, as described above. Specifically, for faces of each race, we predicted mean racial prototypicality ratings (Caucasian-appearance, African-appearance, and Asian-appearance) by White, Black, or Korean perceivers from their mean skin tone ratings and the facial metrics that had discriminated any of the racial groups, controlling face sex, attractiveness, babyfaceness, and smile ratings. The control variables were entered at Step 1. To determine the unique variance accounted for by skin tone, facial metrics were entered at Step 2, and perceived skin tone was entered at Step 3. To determine the unique variance accounted for by facial metrics, perceived skin tone was entered at Step 2 and facial metrics were entered at Step 3. These regression analyses were repeated for faces of each race rated by each of the three groups of perceivers. Table 4 shows the changes in R^2^ associated with the discriminating facial metrics and perceived skin tone when each was entered at Step 3 of the regression analyses predicting the three racial prototypicality ratings of White, Black, and Korean faces by perceivers of each race.

**Table 4:** Contribution of Skin Tone and Facial Metrics to Racial Prototypicality of White, Black, and Korean Faces.^a^

	Caucasian Prototypicality			African Prototypicality			Asian Prototypicality		
Perceiver Race	White	Black	Korean	White	Black	Korean	White	Black	Korean
	β	β	β	β	β	β	β	β	β
**White Faces**									
Skin Tone ΔR^2^	.04	.20**	.34**	.06*	.13**	.30**	.00	.17**	.21**
Facial Metrics ΔR^2^	.26*	.13*	.06	.23	.15	.09	.27	.12	.12
**Black Faces**									
Skin Tone ΔR^2^	.02	.31**	.14**	.00	.40**	.19**	.00	.26**	.12**
Facial Metrics ΔR^2^	.51**	.20**	.27*	.32*	.19**	.17	.16	.20	.18
**Korean Faces**									
Skin Tone ΔR^2^	.00	.02	.07**	.16**	.13**	.17**	.03	.01	.01
Facial Metrics ΔR^2^	.37**	.33**	.27**	.32**	.26*	.14*	.48**	.39**	.44**

a *Note*. Facial attractiveness, babyfaceness, smile scores, and face sex were controlled in all regressions; metrics also were controlled in the regressions predicting from skin tone, and skin tone was controlled in the regressions prediction from metrics.

* *p*<05;

** *p*<001.

We also performed exploratory analyses to determine the particular facial metrics that influenced perceived racial prototypicality and whether these varied with face and perceiver race. Because we had no a priori predictions for many of the metrics we examined, the large number of metric - prototypicality relationships capitalizes on chance (3 face race × 3 perceiver race × 3 prototypicality ratings × 10 discriminating metrics). We therefore report these data in Supplementary Table S1 for the interested reader rather than in the text.

#### Caucasian prototypicality ratings

Skin tone did not produce a significant change in R^2^ for White perceivers’ ratings of faces of any race, all *ps* >05. In contrast, skin tone produced a significant change in R^2^ for Black and Korean perceivers ratings of White faces and Black faces, and for Korean perceivers ratings of Korean faces, all *p*s <001.

Facial metrics produced a significant change in R^2^ for both White and Black perceivers’ Caucasian prototypicality ratings of White faces, both *ps* <05, as well as Black and Korean faces, all *ps* <001. Facial metrics did not produce a significant change in R^2^ for Korean perceivers’ ratings of White faces, *p*>05, whereas there was a significant change for their ratings of Black faces, *p<05,* and Korean faces, *p*<001.

#### African prototypicality ratings

Skin tone produced a significant change in R^2^ for White perceivers’ ratings of White faces, *p*<05, and Korean faces, *p*<001, but not Black faces, *p*>05. For Black and Korean perceivers, skin tone produced a significant change in R^2^ for African prototypicality ratings of faces of all races, all *p*s <001.

Facial metrics did not produce a significant change in R^2^ for White perceivers’ African prototypicality ratings of White faces, *p*>05, but there was a significant change for their ratings of Black faces, *p*<05, and Korean faces, *p*<001. A similar pattern was shown for Black perceivers, with facial metrics producing a significant change in R^2^ for African prototypicality ratings of Black faces, *p*<001, and Korean faces, *p*<05, but not White faces, *p*>05. For Korean perceivers, facial metrics produced a significant change in R^2^ for African prototypicality ratings of Korean faces, *p*<05, but not White or Black faces, *ps* >05.

#### Asian prototypicality ratings

Skin tone did not produce a significant change in R^2^ for White perceivers’ ratings of White, Black, or Korean faces, all *ps* >05, In contrast, skin tone produced a significant change in R^2^ for Black and Korean perceivers ratings of White and Black faces, *p*s <001, but not Korean faces, *p*>05.

Facial metrics did not produce a significant change in R^2^ for Asian prototypicality ratings of White or Black faces by perceivers of any race, all *ps >05*, whereas the change in R^2^ was significant for Asian prototypicality ratings of Korean faces for perceivers of all races, all *p*s <001.

## Discussion

Within race variations in racial prototypicality have been shown to influence important social outcomes [1], [4], [5]. We add to this literature by determining the relative contribution of skin tone and facial metrics to variations in perceived racial prototypicality by White, Black, and Korean pereceivers, information that is essential to predicting who will experience racial prototypicality biases and from whom. As predicted, the racial prototypicality ratings of White perceivers were the least responsive to variations in skin tone. Across the three prototypicality ratings made for faces of each of the three races, only 2 skin tone effects attained statistical significance for White perceivers, as compared with 7 and 8 significant effects of skin tone for Black and Korean perceivers, respectively. Moreover, White perceivers showed less responsiveness to skin tone than to facial metrics (2 significant effects for skin tone vs. 6 significant effects for facial metrics), whereas Black perceivers showed more responsiveness to skin tone than to metrics (7 vs. 4 significant effects), and Korean perceivers showed fairly high responsiveness to both skin tone and metrics (8 vs. 6 significant effects). In addition, the relative impact of skin tone and facial metrics on racial prototypicality ratings varied with face race. For White faces, skin tone had a more consistent effect than facial metrics (7 significant effects for skin tone vs. 2 significant effects for facial metrics). For Korean faces, skin tone had a less consistent effect than metrics (4 vs. 9 significant effects). In the case of Black faces, the relative influence of skin tone and metrics was fairly equal (6 vs. 5 significant effects), but the pattern varied across perceiver race. For both Black and Korean perceivers, the skin tone effects on all three racial prototypicality ratings were significant, whereas none was significant for White perceivers. In contrast, facial metrics had significant effects on 2 out of 3 racial prototypicality ratings of Black faces for White and Black perceivers, as compared with none for Koreans.

The finding that White perceivers’ racial prototypicality ratings were less responsive to skin tone than to facial metrics is a striking effect given that skin tone and racial prototypicality ratings were both rated by perceivers, whereas facial metrics were objectively assessed. This ascendance of facial metrics is consistent with previous research that studied White perceivers and found that facial metrics trumped skin tone when assessing neural indicators of the emotional salience of faces [11], evaluations of criminals in simulated news reports [12], and prototypicality ratings of racially ambiguous morphs [13]. The finding that White perceivers’ racial prototypicality ratings were less responsive to skin tone than were ratings by Black or Korean perceivers is consistent with the cultural injunction to be ‘color blind,’ suggesting that White Americans may ignore skin tone variations so as not be perceived as racist. Although one could argue that perceivers should also strive to be ‘racial feature blind,’ research indicates that people are largely unaware of the influence of these subtle features, and are also unable to monitor their use, even when instructed to do so [5].

In addition to a possible contribution of culturally variable political correctness to the moderating effects of perceiver race, variations in perceptual experience may also make a contribution. Indeed, Black perceivers ratings of the skin tone of Black faces showed the highest variability (Table 2), suggesting that skin tone may have had stronger effects on prototypicality ratings by Black than White Americans because the former are culturally sensitized to subtle variations in skin tone [10]. However, Korean perceivers did not show more variability in skin tone ratings than White Americans even though their prototypicality ratings did show stronger effects of skin tone. Whatever the explanation for variations across perceiver race, the present findings provide an important qualification to the Brooks and Gwinn conclusion that facial feature variations have a greater effect than skin tone on perceived racial prototypicality [13]. While our data support that claim for White perceivers, they do not support it for others, and the failure to find effects of skin color manipulations in previous research may reflect the focus on White perceivers.

Some limitations to the predictors of racial prototypicality that we have documented should be noted. One is that the facial metrics we assessed were not exhaustive. Neither was our measure of skin tone, and it might be interesting for future research to see whether other qualities, such as hue, pigmentation, and contrast affect prototypicality ratings since they have been found to influence other judgments of faces [24]–[31]. Another limitation is that the faces we used were sampled from particular populations. South Asian faces are different from the East Asian Korean faces we used, and Black faces from various origins are different from the African-American faces we used. However, since the Korean faces were randomly selected from a college yearbook, our racial prototypicality findings should generalize to that population. Moreover, African-American faces, including the particular ones in our sample, have shown socially significant effects of racial prototypicality in previous research [1], [3], [4], which underscores the value of determining what influences their perceived prototypicality. Nevertheless, replication of our findings with other samples of faces is important for assessing their generalizability.

Finally, there were variations in the photographic qualities of the facial images due to our use of a variety of databases in order to secure a sufficient number of target images of each race. However, we do not believe that these compromise our conclusions. First, we divided each facial metric by inter-pupil distance, a standard normalization technique to control for variations in distance from the camera [20]–[22]. Second, although the different data bases made it difficult to obtain comparable objective measures of skin tone and also could have influenced the subjective ratings we used, there was strong inter-rater agreement for these ratings. For our purposes, the contribution to prototypicality judgments of these reliably *perceived* differences in skin tone among faces that are all the same race is more important than any objective measure of differences in skin tone. Moreover, it is important to note that both skin tone and prototypicality ratings were made with reference to faces of a single race. Consequently, between race differences in photographic qualities do not compromise our conclusions regarding the determinants of racial prototypicality within faces of each race, and within-race variations in quality do not compromise our conclusions regarding differences in determinants across perceivers of different races.

Another issue regarding the generalizability of our results is whether they would hold true in more ecologically valid contexts. Some affirmative evidence is provided by the fact that both appearance ratings and facial metrics of static photographs predict trait impressions of dynamic images of the same faces, and they do so even when vocal cues are provided [32], [33]. Moreover, racial prototypicality judgments and other subjective impressions of static facial images, including some used in the present study, predict variations in actual life outcomes among the individuals depicted, variations in photographic qualities notwithstanding [3], [4], [34], [35]. These findings provide reason to believe that the facial metric predictors of prototypicality ratings in the present study would generalize to perceived prototypicality in real life contexts.

The variations in perceived racial prototypicality that we have documented have interesting implications for other race-related responses, including the well-documented other-race effect (ORE), whereby perceivers show poorer recognition of other-race than own-race faces [36]. For example, the stronger influence of skin tone on Black than White perceivers’ prototypicality ratings suggests that large variations in skin tone among faces would reduce the ORE effect more for Black perceivers than for White perceivers. Similarly, the stronger influence of facial metrics on White than Black perceivers’ racial prototypicality ratings suggests that large variations in facial metrics would reduce the ORE effect for White perceivers more than for Black perceivers. This possible contribution of specific facial qualities to the ORE provides a novel addition to accounts that focus on greater experience processing own-race faces or greater motivation to do so [37].

Variations in perceived racial prototypicality also have significant implications for predicting the facial qualities that make people vulnerable to prejudice – those who will experience racial prototypicality biases and from whom, Consider, for example, the evidence that U.S. judges, who are largely White, give longer prison terms to more African-looking White or Black defendants [3], and are more likely to give the death penalty to more African-looking Black defendants convicted of murdering a White victim [4]. Our results indicate that the more African-looking White convicts are those with darker-skin more so than those with more African-looking facial features. Indeed, the greater importance of skin tone than facial metrics when judging African prototypicality of White faces held true in our study regardless of perceiver race. In the case of Black convicts, our results suggest that those with more African-looking features are at greater risk for harsh punishment than those with darker skin if they are sentenced by White perceivers, for whom facial metrics had more impact on African prototypicality ratings. In contrast, if perceivers are Black, then darker skin will have more impact on perceived African prototypicality than more African-looking features. However, there is reason to expect that Black perceivers would not respond negatively to a more African-looking appearance, and they may even respond positively [15], [38], [39]. Finally, for Korean convicts, our results indicate that either darker skin or more African-looking features could put them at greater risk regardless of the perceiver’s race.

Although we have illustrated the implications of our findings for people of different races with a more prototypically African appearance, it would be interesting to explore the implications for people with a more prototypically Asian appearance, particularly given evidence that Asians are viewed as the ‘model minority’ in the United States [40], [41], as well as for people with a more prototypically White appearance. Also, although we have discussed implications for the judicial system, racial prototypicality effects may be found in other domains where facial appearance has been shown to influence social outcomes, including education, employment, and health care [42]. Knowing what objective facial qualities influence subjective racial prototypicality assessments by various perceivers is important for efforts to ameliorate such biases.

## Supporting Information

Table S1 **ΔR^2^ and Standardized Beta Weights from Regressions Predicting Caucasian, African, and Asian Prototypicality.**
(DOC)
