# Supplementary material for: Skin and Bones: The Contribution of Skin Tone and Facial Structure to Racial Prototypicality Ratings
Source: PLoS One. 2012 Jul 18;7(7):e41193. doi: 10.1371/journal.pone.0041193 (PMC3399873; doi:10.1371/journal.pone.0041193)
Supplement: Table S1 — ΔR2 and Standardized Beta Weights from Regressions Predicting Caucasian, African, and Asian Prototypicality. (DOC) [file pone.0041193.s001.doc]

**Table S1**

**ΔR^2^ and Standardized Beta Weights from Regressions Predicting Caucasian, African, and Asian Prototypicality**

| Perceiver Race | White | Black | Korean |
| --- | --- | --- | --- |
| Caucasian Prototypicality | β | β | β |
| **White Faces** |  |  |  |
| **Skin Tone ΔR^2^** | **.04+** | **.20**** | **.34**** |
| **All facial metrics ΔR^2^** | **.26*** | **.13*** | **.06** |
| Eyebrow Height B2 | -.04 | .10 | -.04 |
| Eyebrow Separation B1 | .37** | .18+ | .03 |
| Eye Height E5 | .08 | .04 | .08 |
| Eye Separation E7 | .03 | -.10 | -.07 |
| Eye Width E4 | -.21 | -.13 | -.07 |
| Nose Length N3 | .23 | .24* | .19+ |
| Nose Width N2 | -.13 | -.26* | -.11 |
| Lip Thickness M1 | -.01 | -.14 | -.03 |
| Mouth Width M0 | -.04 | .07 | -.05 |
| Chin to Pupil C1 | .22 | .01 | .07 |
| Jaw Width W1 | -.12 | .14 | -.13 |
| **Black Faces** |  |  |  |
| **Skin Tone ΔR^2^** | **.02** | **.31**** | **.14**** |
| **All facial metrics ΔR^2^** | **.51**** | **.20**** | **.27*** |
| Eyebrow Height B2 | .13 | .01 | -.06 |
| Eyebrow Separation B1 | .14 | -.09 | .11 |
| Eye Height E5 | .31* | .05 | .11 |
| Eye Separation E7 | .01 | .18* | .02 |
| Eye Width E4 | -.08 | .10 | .08 |
| Nose Length N3 | .36* | -.00 | .29+ |
| Nose Width N2 | -.57** | -.38** | -.30+ |
| Lip Thickness M1 | -.38** | -.26** | -.25+ |
| Mouth Width M0 | -.02 | -.01 | -.11 |
| Chin to Pupil C1 | .09 | .31+ | .12 |
| Jaw Width W1 | .33* | .03 | .04 |
| **Korean Faces** |  |  |  |
| **Skin tone ΔR^2^** | **.00** | **.02** | **.07**** |
| **All facial metrics ΔR^2^** | **.37**** | **.33**** | **.27**** |
| Eyebrow Height B2 | -.33** | -.34** | -.26* |
| Eyebrow Separation B1 | -.17 | -.20+ | -.17+ |
| Eye Height E5 | .30* | .09 | .19+ |
| Eye Separation E7 | -.18 | -.28* | -.15 |
| Eye Width E4 | .10 | -.00 | -.20+ |
| Nose Length N3 | -.15 | -.18 | .12 |
| Nose Width N2 | .01 | .06 | -.17 |
| Lip Thickness M1 | -.19 | -.16 | -.14 |
| Mouth Width M0 | -.06 | .07 | .12 |
| Chin to Pupil C1 | .17 | .31 | .22 |
| Jaw Width W1 | -.15 | -.22 | -.24+ |

| Perceiver Race | White | Black | Korean |
| --- | --- | --- | --- |
| African Prototypicality | β | β | β |
| **White Faces** |  |  |  |
| **Skin Tone ΔR^2^** | **.06*** | **.13**** | **.30**** |
| **All facial metrics ΔR^2^** | **.23+** | **.15+** | **.09** |
| Eyebrow Height B2 | .02 | -.07 | .09 |
| Eyebrow Separation B1 | -.31* | -.16 | -.02 |
| Eye Height E5 | .09 | .10 | -.08 |
| Eye Separation E7 | -.18 | .13 | .06 |
| Eye Width E4 | .19 | .08 | .18+ |
| Nose Length N3 | -.23 | -.13 | -.31** |
| Nose Width N2 | .17 | .35** | .20* |
| Lip Thickness M1 | -.01 | .10 | .06 |
| Mouth Width M0 | .09 | -.02 | .01 |
| Chin to Pupil C1 | -.12 | -.19 | .15 |
| Jaw Width W1 | -.05 | -.24+ | -.11 |
| **Black Faces** |  |  |  |
| **Skin Tone ΔR^2^** | **.00** | **.40**** | **.19**** |
| **All facial metrics ΔR^2^** | **.32*** | **.19**** | **.17** |
| Eyebrow Height B2 | -.11 | -.01 | .10 |
| Eyebrow Separation B1 | -.07 | .17* | -.04 |
| Eye Height E5 | -.13 | .04 | -.05 |
| Eye Separation E7 | -.09 | -.22* | -.01 |
| Eye Width E4 | -.04 | -.16* | -.05 |
| Nose Length N3 | -.41* | -.06 | -.31* |
| Nose Width N2 | .43* | .31** | .16 |
| Lip Thickness M1 | .33* | .30** | .28* |
| Mouth Width M0 | .14 | -.03 | .09 |
| Chin to Pupil C1 | .18 | -.10 | .13 |
| Jaw Width W1 | -.38* | .02 | -.14 |
| **Korean Faces** |  |  |  |
| **Skin Tone ΔR^2^** | **.16**** | **.13**** | **.17**** |
| **All facial metrics ΔR^2^** | **.32**** | **.26*** | **.14*** |
| Eyebrow Height B2 | -.08 | .06 | -.12 |
| Eyebrow Separation B1 | .08 | .14 | -.11 |
| Eye Height E5 | .11 | .40** | .11 |
| Eye Separation E7 | -.09 | .14 | .24* |
| Eye Width E4 | .35* | .14 | .34** |
| Nose Length N3 | -.24 | -.20 | -.30* |
| Nose Width N2 | .34* | .37* | .07 |
| Lip Thickness M1 | .45** | .14 | .22* |
| Mouth Width M0 | -.03 | -.05 | -.11 |
| Chin to Pupil C1 | -.14 | -.10 | .22 |
| Jaw Width W1 | -.34* | -.25 | -.16 |

| Perceiver Race | White | Black | Korean |
| --- | --- | --- | --- |
| Asian Prototypicality | β | β | β |
| **White Faces** |  |  |  |
| **Skin Tone ( ΔR^2^)** | **.00** | **.17**** | **.21**** |
| **All facial metrics ΔR^2^** | **.27+** | **.12** | **.12** |
| Eyebrow Height B2 | .04 | -.10 | -.03 |
| Eyebrow Separation B1 | -.32* | -.14 | -.03 |
| Eye Height E5 | -.29 | -.18 | -.06 |
| Eye Separation E7 | .20 | .05 | .06 |
| Eye Width E4 | .15 | .14 | -.06 |
| Nose Length N3 | -.13 | -.30* | -.01 |
| Nose Width N2 | .02 | .07 | -.02 |
| Lip Thickness M1 | .04 | .13 | -.01 |
| Mouth Width M0 | -.05 | -.10 | .08 |
| Chin to Pupil C1 | -.27 | .18 | -.30+ |
| Jaw Width W1 | .32+ | .01 | .37* |
| **Black Faces** |  |  |  |
| **Skin Tone ΔR^2^** | **.00** | **.26**** | **.12**** |
| **All facial metrics ΔR^2^** | **.16** | **.20+** | **.18** |
| Eyebrow Height B2 | .04 | -.01 | -.13 |
| Eyebrow Separation B1 | -.04 | -.24+ | -.08 |
| Eye Height E5 | -.12 | -.17 | -.05 |
| Eye Separation E7 | .14 | .18 | -.01 |
| Eye Width E4 | .16 | .20 | -.01 |
| Nose Length N3 | .29 | .15 | .22 |
| Nose Width N2 | -.09 | -.08 | .08 |
| Lip Thickness M1 | -.13 | -.25+ | -.21 |
| Mouth Width M0 | -.20 | .08 | -.04 |
| Chin to Pupil C1 | -.40 | -.30 | -.42+ |
| Jaw Width W1 | .28 | -.11 | .21 |
| **Korean Faces** |  |  |  |
| **Skin Tone ΔR^2^** | **.03+** | **.01** | **.01** |
| **All facial metrics ΔR^2^** | **.48**** | **.39**** | **.44**** |
| Eyebrow Height B2 | .38** | .34** | .40** |
| Eyebrow Separation B1 | .13 | .14 | .29* |
| Eye Height E5 | -.36** | -.34* | -.32* |
| Eye Separation E7 | .23+ | .23 | -.04 |
| Eye Width E4 | -.30* | -.08 | -.07 |
| Nose Length N3 | .28 | .31 | .12 |
| Nose Width N2 | -.20 | -.28* | .14 |
| Lip Thickness M1 | -.06 | .09 | -.03 |
| Mouth Width M0 | .08 | -.05 | -.04 |
| Chin to Pupil C1 | -.09 | -.28 | -.44* |
| Jaw Width W1 | .34* | .39* | .41* |

Note: ΔR^2^  values for facial metrics control skin tone and those for skin tone control facial metrics.

+ *p* < .10*.* * *p < .05. ** p*  < .001
